# On the Mechanism of Gene Silencing in *Saccharomyces cerevisiae*

**DOI:** 10.1534/g3.115.018515

**Published:** 2015-06-16

**Authors:** David Lee Steakley, Jasper Rine

**Affiliations:** Department of Molecular and Cell Biology, California Institute of Quantitative Biosciences, Stanley Hall, University of California Berkeley, Berkeley, California 94720

**Keywords:** heterochromatin, transcription, repression, eukaryotic gene regulation, euchromatin

## Abstract

Multiple mechanisms have been proposed for gene silencing in *Saccharomyces cerevisiae*, ranging from steric occlusion of DNA binding proteins from their recognition sequences in silenced chromatin to a specific block in the formation of the preinitiation complex to a block in transcriptional elongation. This study provided strong support for the steric occlusion mechanism by the discovery that RNA polymerase of bacteriophage T7 could be substantially blocked from transcribing from its cognate promoter when embedded in silenced chromatin. Moreover, unlike previous suggestions, we found no evidence for stalled RNA polymerase II within silenced chromatin. The effectiveness of the Sir protein–based silencing mechanism to block transcription activated by Gal4 at promoters in the domain of silenced chromatin was marginal, yet it improved when tested against mutant forms of the Gal4 protein, highlighting a role for specific activators in their sensitivity to gene silencing.

Transcriptional silencing is a form of regional, promoter-independent repression mediated in *Saccharomyces cerevisiae* by the Silent Information Regulator (SIR) protein complex. Sir proteins are the structural components of a specialized structure of chromatin that is analogous to heterochromatin in other species. Sir protein–based silencing represses transcription of the *HML* and *HMR* loci, which contain auxiliary copies of the genes that specify mating-type identity. Transcriptional silencing results in a 1000-fold reduction in transcript levels of the genes at *HML* and *HMR* compared to expression of those same genes when at the active *MAT* locus. Sir-based silencing also operates on some genes close to yeast telomeres (Aparacio *et al.* 1991; reviewed in Wellinger and Zakian 2012).

The DNA elements and proteins required to establish and maintain silencing have been identified (reviewed in Rusche *et al.* 2003; Kueng et al 2013). Silencing of transcription requires the recruitment of a complex consisting of Sir2, Sir3, and Sir4 to the silencers and *HML* and *HMR*. In addition, silencing also requires the catalytic activity of Sir2, a histone deacetylase acting on acetylated H4K16 (Imai *et al.* 2000; Landry *et al.* 2000), and the assembly of a repressive chromatin domain by interactions among multiple copies of the Sir protein complex throughout the domain (Rusche *et al.* 2002; Thurtle and Rine 2013). The molecular mechanisms by which the binding of the Sir proteins and the establishment of a silenced local chromatin domain repress transcription are the subject of this study.

Three classes of models have been proposed for how Sir proteins silence transcription. A steric occlusion model is inspired by silenced chromatin’s ability to block binding/action of sequence-specific DNA binding proteins, such as HO endonuclease, DNA methylase, and restriction enzymes to their binding sites in silenced chromatin (Singh and Klar 1992; Strathern *et al.* 1982; Gottschling 1992; Loo and Rine 1994). Thus, Sir proteins create a specialized local chromatin structure that exhibits reduced accessibility to site-specific DNA-binding proteins and, by extension, presumably to the transcription machinery. However, the reduced accessibility in these studies was measured qualitatively. Hence, how much of the 1000-fold repression of *HML* and *HMR* expression could be accounted for by this mechanism has been unresolved.

A preinitiation-complex interference model is a refinement of steric occlusion models in response to a possible mismatch between the magnitude of steric occlusion in previous studies and the 1000-fold reduction in transcription in silenced chromatin. The preinitiation-complex interference model is based on chromatin immunoprecipitation (ChIP) analyses indicating the absence of key components of general transcription machinery, TFIIB (Sua7), TFIIE (Tfa2), and RNA Pol II (Rpb1) from silenced chromatin. In contrast, occupancy of Ppr1, the gene-specific activator for a silenced *URA3* transgene, is only slightly reduced in silenced chromatin (Chen and Widom 2005). The preinitiation-complex interference model posits that the reduction in transcription achieved in silenced chromatin results from the sensitivity of specific factors within the preinitation-complex to being blocked from accessing their target sites, preventing the formation of a preinitation-complex.

In contrast to the previous two models, the downstream-inhibition model is inspired by ChIP data interpreted as showing that, in addition to RNA Pol II, components of the preinitation-complex, TBP (Spt15) and TFIIH (Tfb1, Kin28) are comparably enriched in silenced and active chromatin, but mRNA capping proteins (Cet1, Abd1) and downstream elongation factors (Spt5, TFIIS, Paf1) are specifically absent (Gao and Gross 2008). In this model, RNA polymerase II is blocked by Sir proteins at the transition between initiation and elongation. In support of this observation, gene-specific activators and RNA Pol II are reported to be localized to *HSP82* placed adjacent to *HMR-E* (Sekinger and Gross 2001).

To provide greater resolution toward the mechanism of silencing, we performed specific tests of predictions made by these models, asking whether the inferred stalled transcription complex at *HML* exists, and whether the function of a completely heterologous, prokaryotic RNA polymerase can be quantitatively blocked by Sir-based silencing *in vivo*. Finally, we determined whether transcription activators differed with respect to their sensitivity to Sir-based silencing.

## Materials and Methods

### Yeast strain construction and media

All strains used in this study were derived from W303-1a and are listed in Table 1. All plasmids used in this study are listed in Table 2 and oligonucleotides used in this study are listed in Table 3. Standard mating and sporulation techniques were used to perform yeast crosses. The *T7pro*::*a1* allele, consisting of a 20-base pair (bp) optimal T7 promoter, as described by Ujvári and Martin (1997), fused to the wild-type *a1* ORF including 5 bp directly upstream of the ATG to allow for T7 polymerase initiation, was synthesized with 50 bp of homology matching the native *HMRa1* locus upstream and downstream of the *T7pro*::*a1* allele (pJR 3208). The *T7pro*::*a1* allele and homology regions were amplified by PCR using oDS 82 and oDS 83 and transformed into JRY8676. Transformants were counter-selected with 5-FOA. 5-FOA-resistant colonies were screened by PCR, and the structure of the new *HMR* allele was verified by sequencing. All further strains bearing *HMR T7pro*::*a1* were generated by standard mating and tetrad dissection, and segregates were verified by PCR and sequencing to confirm the presence of *T7pro*::*a1*. Strains bearing *GAL1pro*::*a1* alleles were constructed in an identical fashion, with 450 bp of the *GAL1* promoter directly upstream of *HMRa1* (pJR 3209) (Mr. Gene, GmbH, Germany).

**Table 1:** Yeast strains used in this study

Strain	Parent	Genotype	Source	Plasmid
JRY4012	W303	*MATa his3-11 leu2-3*, *112 lys2 trp1-1 ura3-1 can1-100*	R. Rothstein	
JRY4579	W303	*MATa sir4∆*::*TRP1 his3-11 leu2-3*, *112 lys2 trp1-1 ura3-1 can1-100*		
JRY8676	W303	*MAT****α*** *HMRa1ORF*::*K.lactis URA3ORF sir4∆*::*HIS3 ade2 his3 leu2 trp1 ura3*		
JRY9514	W303	*MAT****α*** *HMR:T7pro*::*a1 ade2 his3 leu2 ura3 [pJR3207]*	This study	pJR3207
JRY9515	W303	*MAT****α*** *HMR:T7pro*::*a1 sir4∆*::*HIS3 ade2 his3 leu2 ura3 [pJR3207]*	This study	pJR3207
JRY9516	W303	*MAT****α*** *ade2 his3 leu2 ura3 [pJR3207]*	This study	pJR3207
JRY9517	W303	*MAT****α*** *sir4*∆::*HIS3 ade2 his3 leu2 ura3 [pJR3207]*	This study	pJR3207
JRY9518	W303	*MAT****α*** *HMR:T7pro*::*a1 sir4*∆::*HIS3 ade2 his3 leu2 ura3 [pJR1237]*	This study	pJR1237
JRY9519	W303	*MAT****α*** *HMR:a2/a1promoter*∆ *sir4*∆::*HIS3 ade2 his3 leu2 trp1 ura3*	This study	
JRY9520	W303	*MAT****α*** *HMR:T7pro*::*a1 sir4*∆::*HIS3 URA3:GAL1promoterNLS-T7polymerase ade2 his3 leu2 ura3*	This study	
JRY9521	W303	*MAT****α*** *HMR:T7pro*::*a1 sir4*∆::*HIS3 URA3:GAL1promoterNLS-T7polymerase ade2 his3 leu2 ura3 / MATa his3-11 leu2-3*, *112 lys2 trp1-1 ura3-1 can1-100*	This study	
JRY9522	W303	*MAT****α*** *HMR:T7pro*::*a1 sir4*∆::*HIS3 URA3:GAL1promoterNLS-T7polymerase ade2 his3 leu2 ura3 / mat*∆::*KANMX hmr*∆::*HYGMX hml*∆::*NATMX ade2 his3 leu2 trp1 ura3*	This study	
JRY9523	W303	*MAT****α*** *HMR:T7pro*::*a1 13xMYC-SIR3:KANMX ade2 his3 leu2 trp1 ura3 [pJR3207]*	This study	pJR3207
JRY9524	W303	*MAT****α*** *HMR:T7pro*::*a1 13xMYC-SIR3:KANMX sir4*∆::*HIS3 ade2 his3 leu2 trp1 ura3 [pJR3207]*	This study	pJR3207
JRY9525	W303	*mat*∆::*HYGMX HMR:T7pro*::*a1 13xMYC-SIR3*::*KANMX ade2 his3 leu2 trp1 ura3*	This study	
JRY9526	W303	*MAT* ***α*** *HMR:GAL1pro*::*a1 13xMYC-SIR3:KANMX ade2 his3 leu2 trp1 ura3*	This study	
JRY9527	W303	*MAT* ***α*** *HMR:GAL1pro*::*a1 13xMYC-SIR3:KANMX sir4*Δ::*HIS3 ade2 his3 leu2 trp1 ura3*	This study	
JRY4013	W303	*MAT* ***α*** *his3-11 leu2-3*, *112 lys2 trp1-1 ura3-1 can1-100*	R. Rothstein	
JRY2726		*MATa his4*		
JRY2728		*MAT* ***α*** *his4*		
JRY9528	W303	*MAT* ***α*** *HMR:GAL1pro*::*a1 13xMYC-SIR3:KANMX gal4*Δ::*NATMX ade2 his3 leu2 trp1 ura3 [pJR3210]*	This study	pJR3210
JRY9529	W303	*MAT* ***α*** *HMR:GAL1pro*::*a1 13xMYC-SIR3:KANMX gal4*Δ::*NATMX ade2 his3 leu2 trp1 ura3 [pJR3211]*	This study	pJR3211
JRY9530	W303	*MAT* ***α*** *HMR:GAL1pro*::*a1 13xMYC-SIR3:KANMX sir4*Δ::*HIS3 gal4*Δ::*NATMX ade2 his3 leu2 trp1 ura3 [pJR3210]*	This study	pJR3210
JRY9531	W303	*MAT* ***α*** *HMR:GAL1pro*::*a1 13xMYC-SIR3:KANMX sir4*Δ::*HIS3 gal4*Δ::*NATMX ade2 his3 leu2 trp1 ura3 [pJR3211]*	This study	pJR3211
JRY9743	W303	*MAT* ***α*** *HMR:GAL1pro*::*a1 13xMYC-SIR3:KANMX gal4*Δ::*NATMX ade2 his3 leu2 trp1 ura3 [pJR3376]*	This study	pJR3376
JRY9744	W303	*MAT* ***α*** *HMR:GAL1pro*::*a1 13xMYC-SIR3:KANMX sir4*Δ::*HIS3 gal4*Δ::*NATMX ade2 his3 leu2 trp1 ura3 [pJR3376]*	This study	pJR3376
JRY9745	W303	*MAT* ***α*** *HMR:GAL1pro*::*a1 13xMYC-SIR3:KANMX gal4*Δ::*NATMX ade2 his3 leu2 trp1 ura3 [pJR3377]*	This study	pJR3377
JRY9746	W303	*MAT* ***α*** *HMR:GAL1pro*::*a1 13xMYC-SIR3:KANMX sir4*Δ::*HIS3 gal4*Δ::*NATMX ade2 his3 leu2 trp1 ura3 [pJR3377]*	This study	pJR3377
JRY9747	W303	*MAT* ***α*** *HMR:GAL1pro*::*a1 13xMYC-SIR3:KANMX gal4*Δ::*NATMX ade2 his3 leu2 trp1 ura3 [pJR3378]*	This study	pJR3378
JRY9748	W303	*MAT* ***α*** *HMR:GAL1pro*::*a1 13xMYC-SIR3:KANMX sir4*Δ::*HIS3 gal4*Δ::*NATMX ade2 his3 leu2 trp1 ura3 [pJR3378]*	This study	pJR3378
JRY9749	W303	*MAT* ***α*** *HMR:GAL1pro*::*a1 13xMYC-SIR3:KANMX gal4*Δ::*NATMX ade2 his3 leu2 trp1 ura3 [pJR3379]*	This study	pJR3379
JRY9750	W303	*MAT* ***α*** *HMR:GAL1pro*::*a1 13xMYC-SIR3:KANMX sir4*Δ::*HIS3 gal4*Δ::*NATMX ade2 his3 leu2 trp1 ura3 [pJR3379]*	This study	pJR3379
JRY9751	W303	*MAT* ***α*** *HMR:GAL1pro*::*a1 13xMYC-SIR3:KANMX gal4*Δ::*NATMX ade2 his3 leu2 trp1 ura3 [pJR3380]*	This study	pJR3380
JRY9752	W303	*MAT* ***α*** *HMR:GAL1pro*::*a1 13xMYC-SIR3:KANMX sir4*Δ::*HIS3 gal4*Δ::*NATMX ade2 his3 leu2 trp1 ura3 [pJR3380]*	This study	pJR3380
JRY9753	W303	*MAT* ***α*** *HMR:GAL1pro*::*a1 13xMYC-SIR3:KANMX gal4*Δ::*NATMX ade2 his3 leu2 trp1 ura3 [pJR3381]*	This study	pJR3381
JRY9754	W303	*MAT* ***α*** *HMR:GAL1pro*::*a1 13xMYC-SIR3:KANMX sir4*Δ::*HIS3 gal4*Δ::*NATMX ade2 his3 leu2 trp1 ura3 [pJR3381]*	This study	pJR3381

Unless otherwise noted, strains were from the lab strain collection.

**Table 2:** Plasmids used in this study

Plasmid	Backbone	Bacteria Selection	Yeast Selection	Insert	Source
pJR3207	pUC18	Amp	*LEU2*	GAL1pro:NLS-T7 Polymerase	Benton *et al.* (1990)
pJR1237	pRS425	Amp	*LEU2*	Empty vector	Brachmann *et al.* (1998)
pJR3208	pMA-T	Amp	None	*T7pro*::*a1*	This study, Mr. Gene
pJR3209	pMK-RQ	Kan	None	*GAL1pro*::*a1*	This study, Mr. Gene
pJR2781	pRS41H	Amp	*HYG*	Empty vector	Taxis and Knop (2006)
pJR3210	pRS41H	Amp	*HYG*	*gal4L331P*	This study
pJR3211	pRS41H	Amp	*HYG*	*GAL4*	This study
pJR3376	pRS41H	Amp	*HYG*	*gal4-147*∆*768*	This study
pJR3377	pRS41H	Amp	*HYG*	*gal4-238*∆*768*	This study
pJR3378	pRS41H	Amp	*HYG*	*gal4-763*∆*851*	This study
pJR3379	pRS41H	Amp	*HYG*	*gal4-238*∆*851*	This study
pJR3380	pRS41H	Amp	*HYG*	*gal4-848*∆	This study
pJR3381	pRS41H	Amp	*HYG*	*gal4-844*∆	This study

**Table 3:** Oligonucleotides used in this study

Purpose	Name	Sequence
**LM-PCR**	LMPCR linker A (oDS 35)	GCGGTGATTTAAAAGATCTGAATTC
	LMPCR Linker B (oDS 36)	GAATTCAGATC
	HMLα1-LM-PCR-1 (oDS 37)	TGCTCAGCTAGACGTTTTTC
	HMLα1-LM-PCR-2 (oDS 38)	CGTTTTTCTTTCAGCTTTTTTGA
	HMLα1-LM-PCR-3 (oDS 39)	CAGCTTTTTTGAAACCGCTGTG
**Strain construction**	T7pro::a1/GAL1pro::a1 knock in primer at HMR F (oDS 82)	TTTTTCTGTGTAAGTTGATAATTACTTCTATCGTTTTCTATGCTGCGCAT
	T7pro::a1/GAL1pro::a1 knock in primer at HMR R (oDS 83)	GAAACTAAAAGAAAAACCCGACTATGCTATTTTAATCATTGAAAACGAAT
	GAL4 KO F	ATCATTTTAAGAGAGGACAGAGAAGCAAGCCTCCTGAAAGCGGATCCCCGGGTTAATTAA
	GAL4 KO R	GAAGTGAACTTGCGGGGTTTTTCAGTATCTACGATTCATTCGATGAATTCGAGCTCGTTT
**qPCR**	a1 qPCRr F	TGGATGATATTTGTAGTATGGCGGA
	a1 qPCR R	TCCCTTTGGGCTCTTCTCTT
	ACT1 qPCR F	TGTCCTTGTACTCTTCCGGT
	ACT1 qPCR R	CCGGCCAAATCGATTCTCAA
	ARS504 qPCR F	GTCAGACCTGTTCCTTTAAGAGG
	ARS504 qPCR R	CATACCCTCGGGTCAAACAC
	TEL VIR 1.2 kb qPCR F	GTGCTAAAGGAATCCCCAGAGA
	TEL VIR 1.2 kb qPCR R	TCTGTCCATTTTCCCTCTGCTC
	HMR E qPCR F	CGAACGATCCCCGTCCAAGTTATGAGC
	HMR E qPCR R	CAGGAGTACCTGCGCTTATTCTCAAAC
	HMR I qPCR F	AGTTTCAGCTTTCCGCAACAGT
	HMRa1 3′ qPCR F	CCAACATTTTCGTATATGGCG
	HMRa1 3′ qPCR R	CTTGTGCAAATTCCAACTAAAGG
	HMR a2 C qPCR F	CTTCTATCGTTTTCTATGCTGCG
	GAL1 promoter qPCR F	GAGCCCCATTATCTTAGCCTAAAAAAAC
	GAL1 promoter qPCR R	TACTGCCAATTTTTCCTCTTCATAACC
	GAL1 3′ ORF qPCR F	GAACGAGTCTCAAGCTTCTTGC
	GAL1 3′ ORF qPCR R	GCTGGTTTAGAGACGATGATAGC

The *gal4L331P* allele (gift from M. Johnston) was cloned into pJR 2781 using *Bam*HI and *Hin*dIII digestion and ligation, producing plasmid pJR3210. Wild-type *GAL4* was also cloned into pJR2781 using an identical protocol, resulting in pJR3211. Further *gal4* alleles were cloned by QuikChange site-directed mutagenesis on pJR3211, resulting in pJR3376-pJR3381. The resulting *gal4* alleles were verified by sequencing the entire ORF in both directions to ensure only the desired changes were created.

For galactose induction experiments, cells were grown to OD_600_ ≈ 0.7 in CSM raffinose or CSM-Leu raffinose (2%); then, prewarmed 20% galactose was added to a final concentration of 2%. All solution percentages are wt/vol. Fresh CSM galactose or CSM-Leu galactose media were added to samples for kinetic experiments to maintain culture volume and OD over time. All media in nonkinetic experiments were 2% of the indicated sugar.

### Potassium permanganate transcription bubble assay

**Figure 1:**
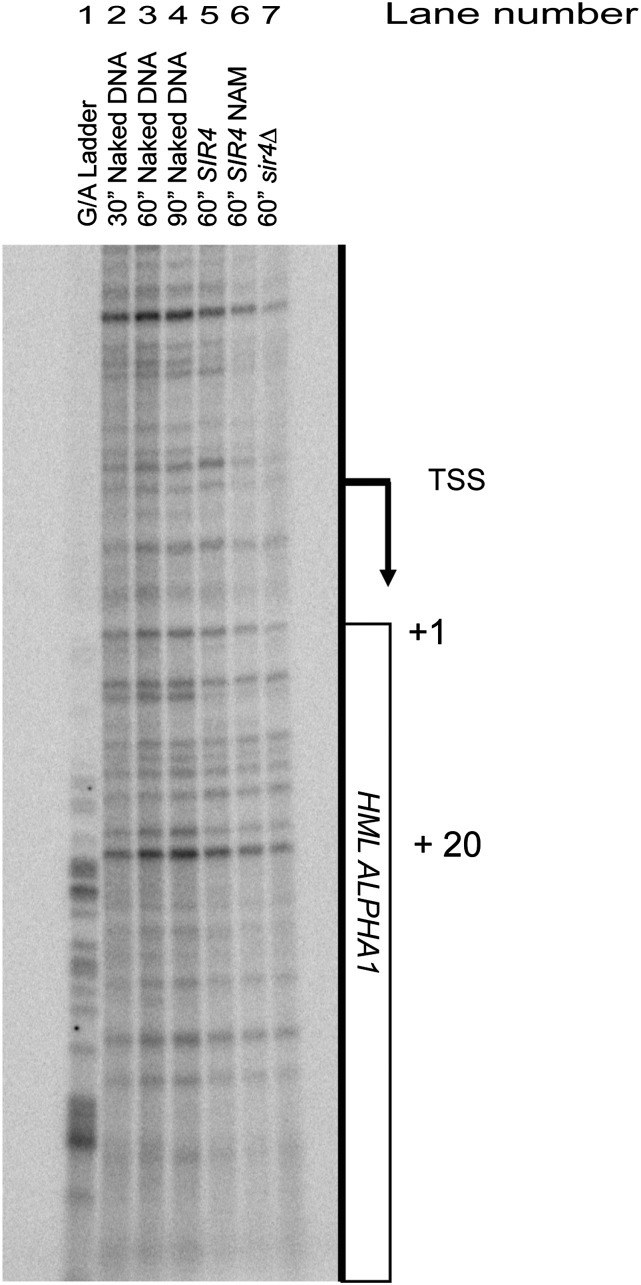
The KMnO_4_ reactivity of *HMLα1 in vivo*. The pattern of KMnO_4_ reactivity is shown for the promoter and 5′ region of *HML****α****1* coding region. Genomic sequences of A+G are shown as a G/A ladder in lane 1. Naked genomic DNA reacted with 20 mM KMnO_4_ for various times is shown in lanes 2–4. The pattern of reactivity of this region in cells reacted with KMnO_4_ is shown in lanes 5–7. The reactivity pattern for cells with *HML****α*** repressed lane 5, cells in which *HML* was derepressed with 5 mM nicotinamide (lane 6) or genetically by *sir4∆* (lane 7) all lack enhanced cleavage sites characteristic of paused/stalled RNA polymerase. The arrow denotes the transcription start site (TSS) (Zhang and Dietrich 2005). The numbers on the right side of the panel identify bases in *HML****α****1* beginning at the initiation ATG codon as +1.

Ten OD units of exponentially growing cells (OD_600_ ≈ 0.9–1) grown in CSM or CSM-5 mM nicotinamide were harvested and resuspended in 1 ml cold PBS. These whole cells or 12 μg genomic DNA were reacted with 20 mM KMnO_4_ on ice for intervals as depicted in Figure 1. The reaction was stopped with an excess of a 20 mM ED (Tris-Cl, 20 mM EDTA, 1% SDS, 0.4 M β-ME stop solution). DNA was extracted from whole cells using glass bead lysis, phenol-chloroform extraction, and sodium-acetate and ethanol precipitation. Both genomic DNA reacted with KMnO_4_ and DNA extracted from KMnO_4_-treated cells was resuspended in TE and stored at 4°. A G/A genomic DNA ladder was generated by 3-min reaction of 5 μg yeast genomic DNA with 95% formic acid. The resulting modified bases in the naked DNA and *in vivo*–treated DNA samples were converted to double-strand breaks using piperidine (Gilmour and Fan 2009). LM-PCR analysis was performed as described by Gilmour and Fan (2009) using HPLC-PAGE–purified linker primers oDS 35 and oDS 36, as well as oDS 37, oDS 38, and oDS 39 to amplify and label *HML****α***1 LM-PCR products. Before resolving and separating LM-PCR products by gel electrophoresis, ODS 39 was labeled using radioactive 10 mCi/ml gamma-^32^P-ATP (Perkin Elmer). Radioactive LM-PCR products were resolved on a 19:1 Acrylamide:Bis-Acrylamide 6% urea sequencing gel. Radioactivity patterns in the gel were visualized by exposure in a phosphor-imager cassette and scanned on a Typhoon scanner (GE Healthcare).

### mRNA extraction, cDNA preparation, and analysis

RNA was purified from cultures at OD_600_ ≈ 0.75 using the Qiagen RNAeasy kit (Qiagen) and on-column DNAse digestion (Qiagen). cDNA was prepared from 2 μg total RNA using random hexamer primers, and in some experiments oligo dT primers, and the Superscript III cDNA synthesis kit (Invitrogen). cDNA was then quantified by qPCR using the Dynamo SYBR green qPCR kit (NEB) and detected on a Stratagene MX3000 quantitative PCR system. All primer sets were normalized to *ACT1* amplification levels. Samples were analyzed in technical triplicate for each of at least three independent RNA preparations.

### Whole-cell extract preparation and immunoblotting

Protein was extracted from cells grown to an OD_600_ ≈ 0.75 using 20% trichloroacetic acid and solubilized in SDS sample buffer. Extracts were fractionated on standard 10% SDS-PAGE gels and transferred to nitrocellulose membranes by wet-transfer at 150 V for 1 hr. Immunoblotting followed standard protocols and blots were imaged on a Li-COR odyssey imager using secondary antibodies conjugated to IR dyes. Antibodies used in immunoblots were anti-T7 RNA polymerase (Millipore 70566) and anti-Pgk1 (Invitrogen 459250).

### Chromatin immunoprecipitation assay

Cells were cross-linked with 1% formaldehyde at OD_600_ ≈ 0.6 to 0.9 for 20 min at room temperature and then quenched with glycine to a final concentration of 300 mM for 5 min at room temperature. Cells were washed twice with cold Tris-buffered saline and lysed with 0.5 mm zirconia beads in FA-lysis buffer (Aparicio *et al.* 2005) with protease inhibitors (Roche) in a MP Fastprep-24. Chromatin was isolated as described (Aparicio *et al.* 2005). For 13XMyc-Sir3 immunoprecipitations, 50 μl of anti-Myc agarose beads (Sigma E6654) were incubated overnight at 4° with sonicated chromatin from 42.5 ml of culture. For Gal4 immunoprecipitations, 4 μl of anti-Gal4 antibody (Abcam 1396) and 25 μl protein A sepharose (GE Healthcare) were incubated with sonicated chromatin under identical conditions. Resin washes, elution, and DNA purification were performed as described (Aparicio *et al.* 2005). Precipitated DNA fragments were analyzed by qPCR, as described previously. The negative-control primer set for 13XMyc-Sir3 ChIP, *ARS504*, was chosen because it gave a consistently low IP/Input signal indistinguishable from a no-tag control and has been shown not to be bound by Sir3. Gal4 ChIP was normalized to a region downstream of the *GAL1* gene not bound by Gal4. ChIP values are presented as enrichment relative to a control locus [(IP(primer)/IN(primer)]/[IP(control)/IN(control)].

## Results

### No evidence of a stalled polymerase at *HML*

In larger eukaryotes, expression of some genes is regulated by transcriptional pausing in which RNA polymerase engages a promoter, separates the strands, and produces a short transcript before stopping, awaiting a signal that restarts transcription (reviewed in Adelman and Lis 2012). Others have reported, and we have confirmed, that a ChIP experiment with an antibody against RNA polymerase II indicates a detectable level of recovery of *HML* and *HMR* from Sir+ cells above background levels (Gao and Gross 2008). However, we have previously reported various sources of artifacts of ChIP signals. In this case, the relative resistance of silenced chromatin to shearing (Teytelman *et al.* 2009) results in the precipitated chromatin being contaminated with larger flanking DNA carrying adjacent active genes. Hence, ChIP is prone to overestimate the occupancy of silenced genes with factors typically associated with transcription.

The most definitive hallmark of a gene with a paused polymerase is a transcription bubble at a specific position detected by the reactivity of the unpaired bases in the bubble to potassium permanganate (Zeitlinger *et al.* 2007). To date, there has been no example of a canonical transcriptionally paused polymerase in *Saccharomyces* detected by a permanganate assay. However, the ChIP data in support of the downstream-inhibition model suggested that the first transcription factors in the progression of gene activation, which were missing from silent chromatin but present in euchromatin, are Cet1 and Abd1, members of the mRNA capping checkpoint (Gao and Gross 2008). These data would imply that RNA polymerase has assembled the preinitiation complex in silenced chromatin and is stalled on melted DNA strands, awaiting the action of Cet1 and Abd1 to move into productive elongation. If so, then evidence of that stall should be detected by the potassium permanganate assay for paused polymerases.

Potassium permanganate preferentially reacts with and modifies T residues in single-stranded DNA (Rubin and Schmid 1980) and can be used to detect stalled RNA polymerases that have melted the DNA template and opened a characteristic transcription bubble (Giardina *et al.* 1992). Positions of greater or lesser permanganate reactivity are revealed by piperidine cleavage of the reacted DNA and electrophoretic separation of the resulting fragments. The T positions in melted DNA show up as positions of enhanced cleavage relative to naked double-stranded DNA. No positions of increased cleavage, characteristic of a stalled RNA polymerase, were detected anywhere in the upstream or early region of *HML****α****1* (Figure 1, lanes 2, 3, and 4 *vs.* lane 5). Similarly, there was no significant increase in reactivity, relative to the signal in naked DNA, in samples from cells treated with nicotinamide (NAM), a chemical inhibitor of Sir2, or in samples from a *sir4∆* mutant (Figure 1, lanes 2, 3, and 4 *vs.* lane 7). The 5 mM NAM was sufficient for complete derepression of *HMRa1*. Cells grown in 5 mM NAM showed comparable levels of *HMRa1* expression to *sir4∆* cells (data not shown). The absence of evidence of separated DNA strands, which would be required of an engaged RNA polymerase, was inconsistent with the mechanism of silencing operating after transcription initiation.

The recent genome-wide datasets on ChIP-exo-seq of preinitiation-complexes and nascent transcript sequencing, performed in *MATa* cells, allowed independent evaluation of nascent transcripts from *HML****α*** (Churchman and Weissman 2012; Rhee and Pugh 2013). We found no evidence of RNA polymerase binding or early elongation at *HML* in those datasets.

### T7 RNA polymerase was repressed by Sir proteins at *HMR*

The difference between the steric occlusion model and the preinitiation-complex interference model hinges on whether the restriction of access of a protein to its recognition sequence in silenced chromatin is sufficient to account for the 1000-fold repression of silenced chromatin. Moreover, to date, evidence of the steric occlusion model rests on the ability of simple DNA-binding proteins, with no source of energy from nucleotide hydrolysis, to access and modify their target site within silenced chromatin *vs.* euchromatin. One could argue that it is easier to inhibit a simple binding reaction than it is to inhibit a process powered by nucleotide hydrolysis and, hence, the steric occlusion model rests on inadequate tests. Conversely, the preinitiation-complex inhibition invites speculation that key components of the core transcription apparatus are unexpectedly sensitive to occlusion, relative to the transcription factor Ppr1, perhaps having evolved sensitivity to the effect of the Sir proteins.

**Figure 2:**
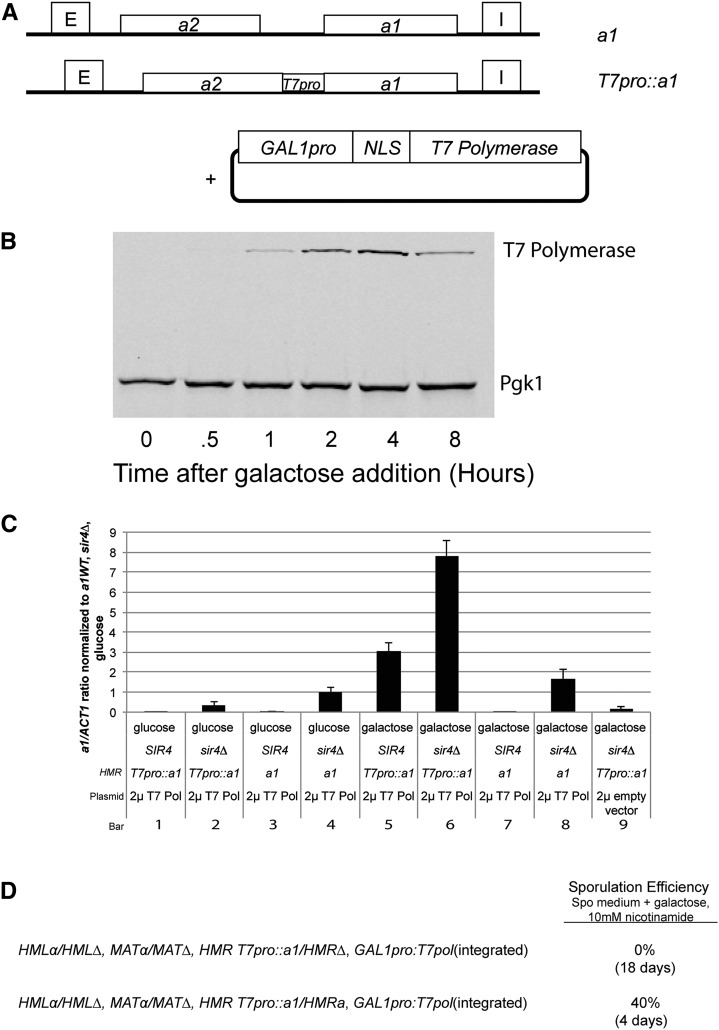
Quantifying transcription and translation of a1 transcripts from *HMR T7pro*::*a1*. (A) A schematic of *T7pro*::*a1* at *HMR* in comparison to wild-type *HMRa1*. Twenty bp of the T7 minimal optimum promoter (Ujvári and Martin 1997) replaced the region between *a2* and 5 bp upstream of the initiation codon for *a1* at *HMR*. The schematic of the 2 μ plasmid (pJR3207) carrying the nuclear localization signal–enhanced T7 RNA polymerase gene is also shown. (B) Protein immunoblot of T7 RNA polymerase protein levels before and upon galactose induction in CSM-leu medium. Pgk1 levels served as a loading control. (C) Quantitation of *a1* transcripts as determined by qRT-PCR of wild-type *a1* and *T7pro*::*a1* at *HMR* in *SIR4* and *sir4*∆, with or without T7 RNA polymerase. All *a1* expression values were normalized to *ACT1* mRNA from the same sample, and that ratio was further normalized to the *a1/ACT1* mRNA ratio in *HMRa1 sir4*∆ strains grown in glucose. All cultures were seeded from saturated overnight growth in the indicated media. cDNA synthesis was primed using random hexamers. Each bar represents the average and SE of three biological replicates. (D) Diagram of strain genotypes used to test possible translation of *a1* transcripts made by T7 RNA polymerase. The top line shows a diploid whose only source of *a1* mRNA would be transcribed from *T7pro*::*a1* by T7 RNA polymerase. The bottom line shows an isogenic control diploid with a source of wild-type *a1* mRNA at *HMR*. Ten mM nicotinamide was used to derepress *HML* and *HMR* and allow for expression of mating-type information normally silenced by Sir proteins. Sporulation requires both *a1* and ***α***2 proteins and the formation of a heterodimer to proceed. Sporulation efficiency was measured as a percentage of cells that formed tetrads.

To challenge the steric occlusion model more rigorously, and to determine whether the yeast core transcription machinery is preternaturally sensitive to blockage by silenced chromatin, we tested whether the RNA polymerase from bacteriophage T7, a single subunit RNA polymerase that evolved independently of native eukaryotic chromatin, was able to transcribe a silenced template. For this experiment, the native promoter of *HMRa1* was replaced with a minimum optimal T7 promoter (*HMR T7pro*::*a1*), as defined by Ujvári and Martin (1997), in cells with a plasmid-encoded, galactose-inducible T7 RNA polymerase endowed with a nuclear localization sequence (Figure 2A). Such a T7 RNA polymerase is able to transcribe a euchromatic locus in yeast (Benton *et al.* 1990).

To quantify the amount of T7 RNA polymerase produced per cell over time, we performed immunoblotting experiments with an antibody against T7 polymerase in a time course following induction in galactose-containing medium (Figure 2B) and compared the signal to that from a dilution series of purified T7 polymerase. T7 RNA polymerase protein levels increased over time, reaching a peak by 4 hr, followed by a gradual decline to steady-state levels. We estimate that the number of T7 polymerase molecules per cell at steady state was approximately 10,000 (data not shown). This value is approximately equal to the number of active RNA Pol II complexes per cell, although there was only one promoter in the genome for T7 RNA polymerase compared to the thousands of promoters for RNA polymerase II (Borggrefe *et al.* 2001).

Upon induction, T7 RNA polymerase was able to transcribe *T7pro*::*a1* at more than five-fold the level of wild-type *HMRa1* in *sir4∆* cells (Figure 2C, bar 6 *vs.* 8). Transcription induced from the *T7pro*::*a1* required T7 RNA polymerase (Figure 2B, bars 5, 6 *vs.* bars 1, 2, 9), although a background level of a1 transcripts, approximately 10% the level from a wild-type *HMRa* locus in *sir4∆* strains, was detected in cells lacking T7 RNA polymerase (Figure 2C, bars 2, 9). A low background level of transcripts was also detected in *sir4*∆ strains deleted for the entire promoter region between *a1* and *a2* of *HMR* (data not shown). Transcription by the T7 RNA polymerase was specific to the T7 promoter: there was no significant increase in *a1* transcription from the *a1* promoter at *HMR* (Figure 2C, bar 4 *vs.* bar 8). At steady state, T7 RNA polymerase was able to transcribe *HMR T7pro*::*a1* in *SIR4* cells at approximately 40% of the level in *sir4∆* cells (Figure 2C, bar 5 *vs.* bar 6).

Some fraction of the *T7pro*::*a1* transcripts were apparently 3′ end-processed and polyadenylated based on their ability to be primed for cDNA synthesis by oligo dT primers, although random hexamers primers detected more transcripts. Previous work has also detected polyadenylated transcripts produced by T7 RNA polymerase in yeast (Sathyanarayana *et al.* 1999). To test whether the transcripts produced by T7 RNA polymerase were translated, we created diploid strains in which the only source of *a1* messages was at *HMR T7pro*::*a1*. Sporulation requires a1 protein and can proceed with even small amounts present (reviewed in Piekarska *et al.* 2010). Such strains were not able to sporulate (0 tetrads among thousands surveyed) in sporulation medium containing galactose and 10 mM nicotinamide to derepress *HML* and *HMR*. In contrast, 40% of cells from an isogenic diploid strain bearing wild-type *HMRa1* incubated in identical medium sporulated (Figure 2D). Therefore, the *T7pro*::*a1* transcripts were not translated into functional a1 protein. Moreover, these data indicated that the background level of T7 polymerase-independent transcripts in *T7pro:a1* in *sir4*∆ strains also did not produce functional a1 protein. Previous work indicates that 5′-transcript capping is defective on T7 transcripts in yeast, which may have provided the block to *a1* translation in our assays (Pinkham *et al.* 1994; Dower and Rosbash 2002).

**Figure 3:**
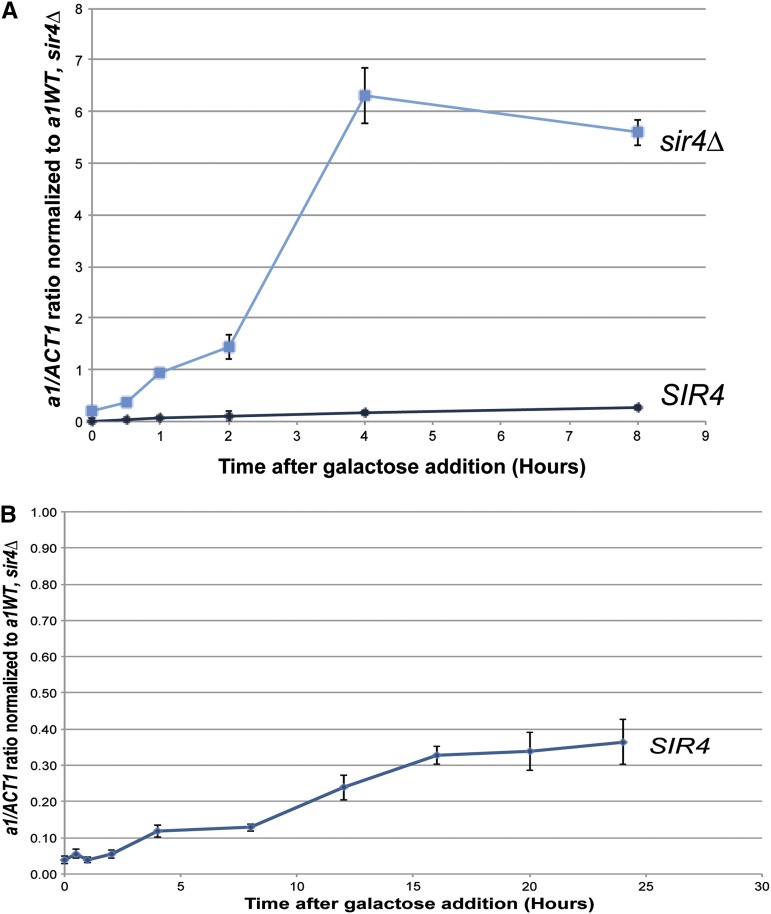
Quantitative transcript analysis of *HMR T7pro*::*a1* upon induction of T7 RNA polymerase. (A) *a1* transcript levels from *T7pro*::*a1* as determined by qRT-PCR in *SIR4* (JRY9523) and *sir4*∆ (JRY9524) strains upon induction of T7 RNA polymerase and media switch from noninducing (raffinose) to inducing (galactose) carbon sources. All *a1* expression values were normalized as in Figure 2 except for galactose cultures being the reference. All cultures were seeded from saturated overnight growth in the indicated media. cDNA synthesis was primed using random hexamers. Each bar represents the average and SE of three biological replicates. (B) *a1* transcript levels from *T7pro*::*a1* as determined by qRT-PCR in *SIR4* cells over an extended induction of T7 RNA polymerase. Note that even at the late time points the level of *a1* expression in *SIR4* cells has still not achieved the same level as in cells chronically grown in galactose medium as in Figure 2. The levels of *a1* expression in the *sir4∆* cells were consistent with the values in Figure 2.

**Figure 4:**
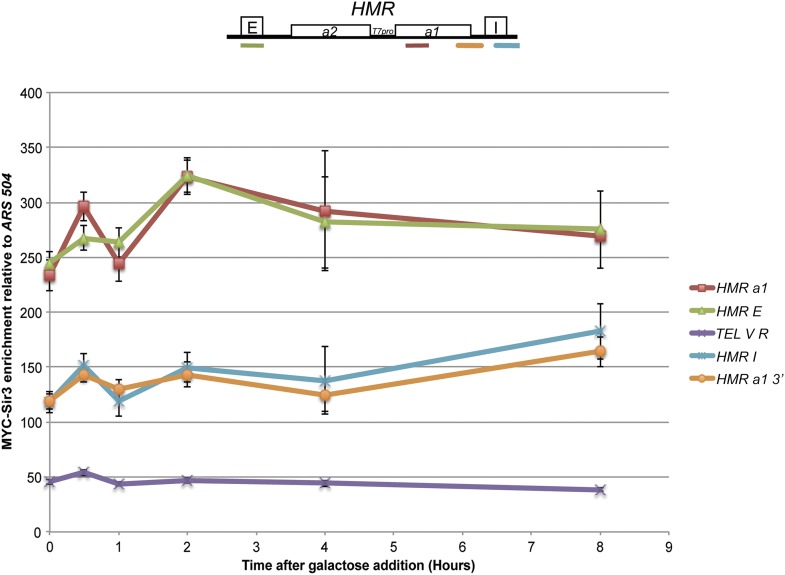
13xMYC-Sir3 enrichment at *HMR* and telomere V R upon induction of T7 RNA polymerase in *SIR4* strains. 13xMYC-Sir3 enrichment as assayed by ChIP followed by qRT-PCR at *HMR* and telomere V R from the same cultures used in Figure 3. Values are displayed as 13xMYC-Sir3 enrichment at the color-coded positions relative to an *ARS504* negative control. The cartoon above the plot shows the location of the primer sets at *HMR*. Each point represents the average and SE of three biological replicates, except the *HMRa1* 8-hr time point, which is an average of two biological replicates.

The ability of T7 RNA polymerase to transcribe from its single promoter at *HMR* in cells that had been grown for many generations with a vast excess of that polymerase was interesting, but not particularly comparable to the challenge faced by RNA polymerase II in trying to transcribe *HML* and *HMR*. Therefore, we measured the *HMR T7pro*::*a1* transcript levels in a time course of T7 RNA polymerase induction. As expected, in *sir4∆* cells, T7 RNA polymerase robustly transcribed *T7pro*::*a1* transcripts beginning as early as 30 min after the beginning of induction, peaking at approximately 4 hr (Figure 3A, *sir4∆*). In contrast, transcription by T7 RNA polymerase was barely detectable in *SIR4* strains at 1 hr, and then increased only slightly over the next 7 hr (Figure 3B). At 2 hr of induction, expression in *SIR4* cells was 200-fold less than in the *sir4*∆ mutant. The magnitude of the difference in expression between *SIR4* cells and *sir4∆* cells decreased slightly at later time points, as discussed further below. Nevertheless, these data established that silenced chromatin had a profound ability to inhibit transcription by T7 RNA polymerase within five-fold of the 1000-fold repression by Sir proteins of the native promoters at *HML* and *HMR*. ChIP analysis revealed that Sir3 protein remained bound throughout the *HMRa1* gene and the entire *HMR* locus in *SIR4* cells throughout the 8-hr induction, in further support of the conclusion that silenced chromatin is largely refractory to transcription by T7 RNA polymerase (Figure 4).

To investigate further transcription by T7 polymerase from *T7pro*::*a1* in *SIR4* strains, we performed an additional identical experiment in *SIR4* cells and tracked *a1* expression over a longer time period. We detected a consistent increase in *T7pro*::*a1* transcripts over time (Figure 3B). As previously shown, when cells were assayed after entering stationary phase, *a1* expression levels increased beyond what was seen at the longest time point in our induction experiment (Figure 2C, bar 5).

### The Gal4 activator was largely insensitive to Sir protein silencing

**Figure 5:**
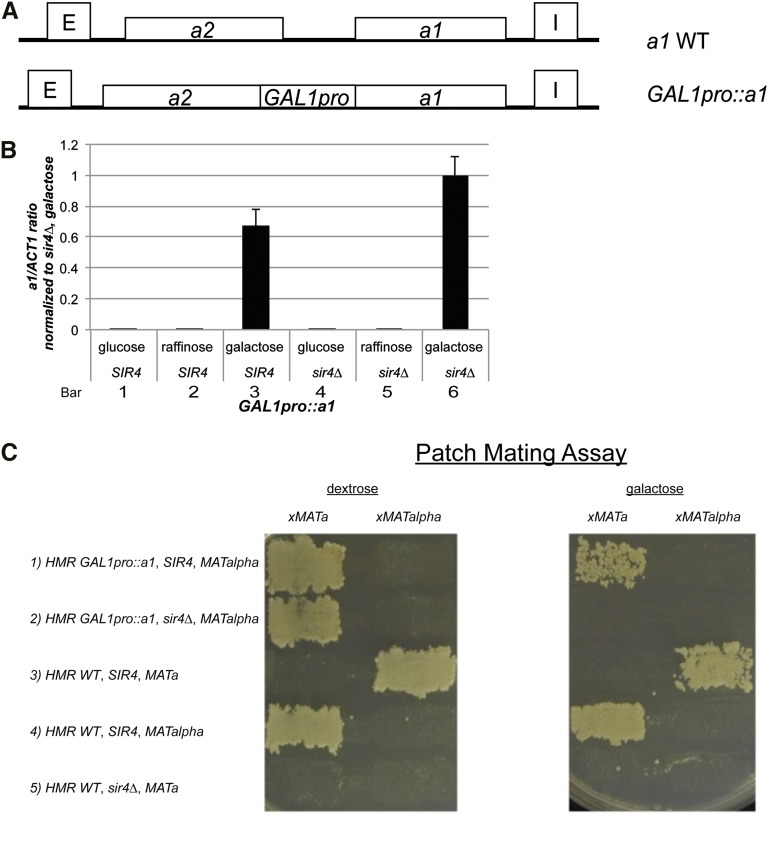
Analysis of expression of *HMR GAL1pro*::*a1*. (A) A schematic of *GAL1pro*::*a1* at *HMR* in comparison to wild-type *a1*. Four hundred fifty bp of the *GAL1* promoter containing the four binding sites of Gal4 (4 UAS_g_ sites) replaced the region between the ORFs of *a2* and *a1* at *HMR*. (B) mRNA expression as determined by qRT-PCR of *GAL1pro*::*a1* at *HMR* in *SIR4* and *sir4*∆ on various carbon sources. All *a1* expression values were normalized to *ACT1* mRNA from the same sample and further to the *a1/ACT1* mRNA ratio in *GAL1pro*::*a1*, *sir4*∆ strains in galactose medium. Each bar represents the average and SEM of three biological replicates. (C) Patch mating assay of various strains with either *HMR GAL1pro*::*a1* or native *HMRa1* on dextrose or galactose media. Query strains were mated to tester strains prototrophic for all markers expect for *his4* and any resulting diploids were replica plated onto minimal medium lacking histidine and containing dextrose or galactose. Growth of diploids demonstrated the ability to mate.

When placed at *HML* or *HMR*, yeast genes vary with respect to their sensitivity to silencing (Brand *et al.* 1985; Chen and Widom 2005; Ren *et al.* 2010). To explore the parameters influencing the sensitivity of a gene to Sir-based silencing, we replaced the promoter of *HMRa1* with the full 450-bp *GAL1* promoter (*HMR GAL1pro*::*a1*), maintaining the approximate size of the *HMR* locus and the distance from the promoter to silencers (Figure 5A). As expected, in *SIR4* and *sir4∆* strains *HMR GAL1pro*::*a1* was not expressed in repressing (glucose medium) or noninducing conditions (raffinose medium) (Figure 5B, bars 1, 2, 4, 5). Again, as expected, *GAL1pro*::*a1* was expressed in *sir4∆* strains grown in galactose medium but not glucose medium (Figure 5B, bar 4 *vs.* 6). In *SIR4* strains grown in inducing medium, *GAL1pro*::*a1* expression was 60% of the level of the *sir4∆* strain (Figure 5B, bar 3 *vs.* bar 6). These results verified that *GAL1pro*::*a1* was expressed at steady state and was largely insensitive to Sir-based silencing.

As expected, the transcripts produced by *GAL1pro*::*a1* were translated based on the a/α diploid mating phenotype of *MAT****α***, *HMR GAL1pro*::*a1*, *sir4∆* strains when grown on galactose-containing medium (Figure 5C, row 2, right-most panel). Likewise, *MAT****α***, *HMR GAL1pro*::*a1*, *SIR4* strains exhibited reduced mating efficiency in cells grown on galactose-containing medium but not in cells grown on glucose-containing medium (Figure 5C, row 1 *vs.* 2).

**Figure 6:**
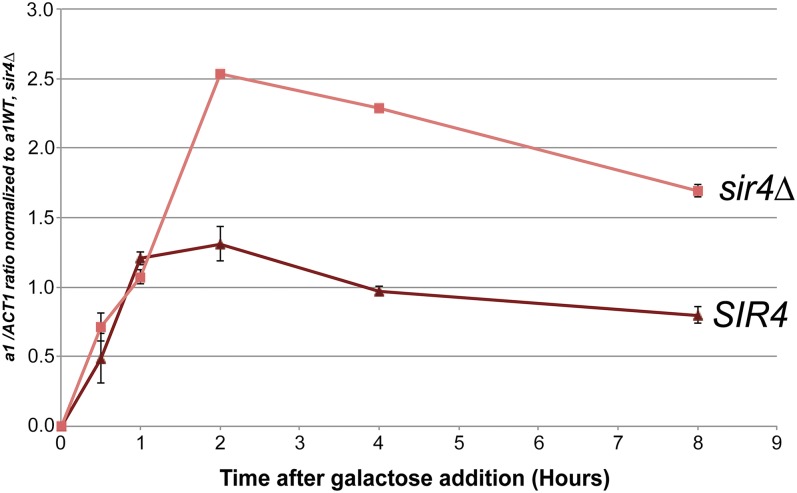
Quantitation of a1 transcripts from *HMR GAL1pro*::*a1* upon induction with galactose in *SIR4* and *sir4∆* strains. *a1* mRNA expression of *GAL1pro*::*a1* as determined by qRT-PCR in *SIR4* and *sir4*∆ strains upon induction with galactose. Strains were initially grown in CSM noninducing (raffinose) medium and galactose was added to induce transcription from the *GAL1* promoter. All *a1* expression values were normalized as in Figure 5. All cultures were seeded from saturated overnight growth in the indicated media. cDNA synthesis was primed using random hexamers. Each bar represents the average and SE of three biological replicates.

Steady-state analyses of the impact of silencing on T7 RNA polymerase obscured the sensitivity to Sir-based silencing that was revealed in kinetic experiments. Hence, we performed analogous kinetic experiments measuring *HMR GAL1pro*::*a1* transcripts in Sir+ and Sir− cells at time points after induction. Remarkably, in the first hour after induction there was no detectable difference in transcription from *HMR GAL1pro*::*a1* between *sir4*∆ cells and *SIR4* cells (Figure 6). After 2 hr, *sir4*∆ strains exhibited approximately two-fold higher levels of *a1* mRNA levels than *SIR4* strains, and this difference in level was relatively unchanged at later time points (Figure 6). The level of *a1* transcripts produced in the *SIR4*, *GAL1pro*::*a1* strain was approximately equal to the level in *sir4∆* strains with a wild-type *HMRa1* locus. Thus, the magnitude of silencing escape of the *GAL1pro*::*a1* allele was significant.

**Figure 7:**
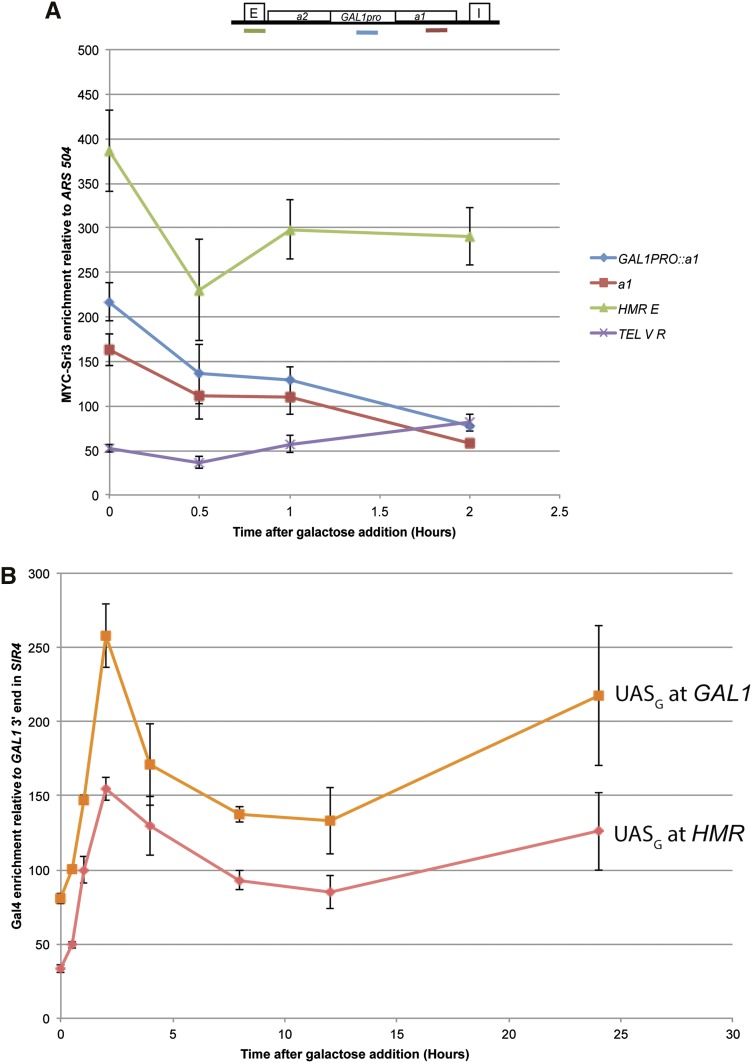
13xMYC-Sir3 and Gal4 enrichment at *HMR GAL1pro*::*a1* upon kinetic galactose induction in *SIR4* strains. (A) 13xMYC-Sir3 enrichment as assayed by Sir3 ChIP followed by qRT-PCR at *HMR* from the identical *SIR4* kinetic cultures described in Figure 6. Values are displayed as 13xMYC-Sir3 enrichment relative to an *ARS504* negative control primer set. All *HMR* primer sets showed a statistically significant reduction in Sir3 occupancy between 0 and 2 hr (see text for p-values). The cartoon above the plot shows the location of the primers sets at *HMR*. Each point represents the average and SEM for three biological replicates. (B) Gal4 enrichment at *HMR GAL1pro*::*a1* and at *GAL1* as assayed by ChIP followed by qRT-PCR. Values are displayed as Gal4 enrichment relative to a negative control primer set mapping to the intergenic region 3′ to the *GAL1* locus. Each point represents three biological replicates and SEM.

Sir protein enrichment at the *GAL1* promoter at *HMR* (*GAL1pro*::*a1*) at an internal region of *a1* and at *HMR-E* significantly decreased between t = 0 and t = 2 hr (paired Student’s *t*-test *P* = 0.012, *P* = 0.009, *P* = 0.021, respectively) (Figure 7A). Although it is possible that transcription caused some loss of Sir proteins from the nucleosomes within the *a1* gene, the reduction at *HMR-E* could not be due to transcription. Despite the considerable transcription of *a1* from the *GAL1* promoter in Sir+ cells (Figure 7A, *SIR4* t = 2), Sir protein enrichment at the *a1* gene remained considerably above background (Figure 7A). We emphasize that at present it is unknown whether Sir proteins occupied the *a1* gene in the same cells that produced a1 transcripts, or whether these assays reflected two different populations in the same culture.

**Figure 8:**
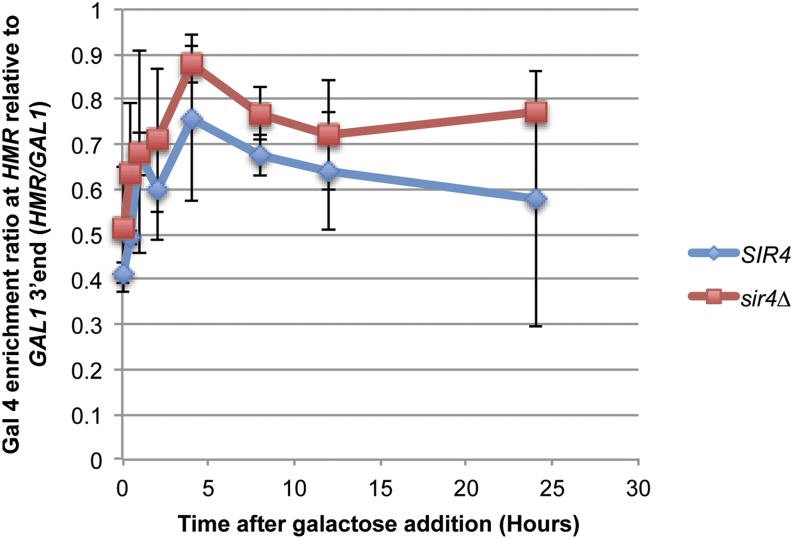
Gal4 enrichment at *HMR GAL1pro*::*a1* and *GAL1* upon galactose induction in *SIR4* and *sir4*∆ strains. Gal4 enrichment as assayed by ChIP followed by qRT-PCR at *HMR* from the same *SIR4* and *sir4*∆ cultures described in Figure 6 and Figure 7. Values are displayed as the ratio of Gal4 enrichment at *HMR GAL1pro*::*a1* relative to enrichment at *GAL1* and a negative control from the intergenic region 3′ to the *GAL1* locus to account for the reduced ability of *HMR GAL1pro*::*a1* to recruit Gal4 compared to native *GAL1*. Each point represents three biological replicates with SEM.

The rapid and identical induction kinetics for transcription in Sir^+^ and Sir^−^ cells raised the possibility that Gal4 protein was bound to the *GAL1 UAS* in (*GAL1pro*::*a1)* prior to induction, just as it is known to bind these sequences at the native *GAL* gene cluster (Lohr and Hopper 1985; Selleck and Majors 1987). Chromatin immunoprecipitation analysis of Gal4 in *SIR4* and *sir4∆* strains revealed that at t = 0, there was considerable enrichment of Gal4 protein at both sites, albeit with two-fold greater enrichment at the native *GAL1* locus (Figure 7B). Upon induction, Gal4 enrichment levels increased at both *HMR GAL1pro*::*a1* and at the native *GAL1* locus, presumably reflecting the well-documented induction of Gal4 protein in galactose medium. At least part of the difference between the enrichment of Gal4 protein at the *GAL1* UAS at the native *GAL* gene cluster *vs.* *HMR* was due to the presence of Sir proteins at *HMR*, as evidenced by comparing the relative enrichment of Sir3 at *HMR* in *SIR4* *vs.* *sir4∆* cells (Figure 8).

**Figure 9:**
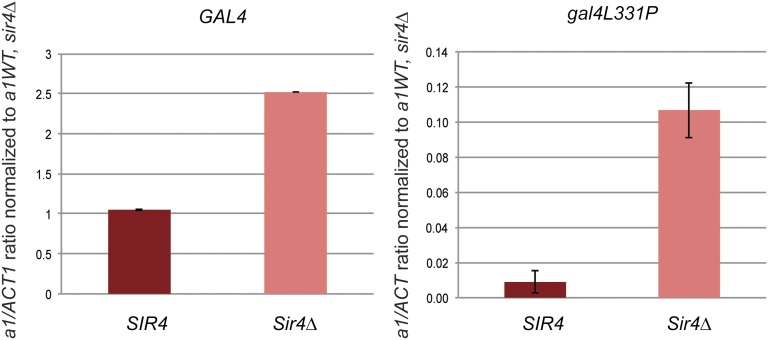
Analysis of *HMR Gal1pro:a1* transcription by wild-type Gal4 *vs.* gal4L331P. a1 mRNA expression from *GAL1pro*::*a1* as determined by qRT-PCR of mRNA from strains bearing Gal4 or the gal4L331P mutant in *SIR4 and sir4∆* in galactose medium. All *a1* expression values were normalized as in Figure 5B. All cultures were seeded from saturated overnight growth. Each bar represents the average and SE of three biological replicates.

Abf1 is the transcription factor responsible for activating transcription from *MATa* (McBroom and Sadowski 1995) and therefore is susceptible to silencing at *HMR*, whose regulatory region sequence is identical to *MATa*. To explore why Gal4 was largely immune from silencing at *HMR* whereas Abf1 is sensitive, we tested whether mutant forms of Gal4 that compromise some aspect of its function would become sensitive to silencing. A point mutant in the central domain of Gal4 (gal4L331P) compromises its ability to activate transcription but not its ability to bind DNA (Johnston and Dover 1988; Mylin *et al.* 1990). As expected, relative to wild-type Gal4, the gal4L331P mutant protein showed a 25-fold reduced ability to activate transcription of *HMR GAL1pro*::*a1* in *sir4∆* strains (Figure 9, note the *y*-axis scale). In *SIR4* cells *a1* transcript levels were even further reduced (Figure 9, right panel), with 12-fold instead of the 2.5-fold reduction of wild-type Gal4. Thus, in this example, a weaker activator exhibited a greater degree of sensitivity to silencing than a stronger version of that same activator. This relationship between sensitivity to silencing and activator strength of Gal4 was observed for some other alleles of *GAL4*, but exceptions such as *gal4-763∆851* indicate that silencing sensitivity is influenced by more than simply activator strength (Table 4).

**Table 4:** Analysis of expression of *HMR GAL1pro*::*a1 in gal4* mutants

Gal4 Allele	DNA Binding Affinity*a* (% of WT)	*GAL1pro*::*a1* mRNA in *sir4∆* as a Percentage of Gal4, *sir4∆*, *GAL1pro*::*a1* mRNA Expression (%)	Ratio of *Gal1pro*::*a1*, *sir4*∆ mRNA */GAL1pro*::*a1*, *SIR* mRNA (*sir4*∆/*SIR*)	*Gal1pro*::*a1*, *sir4∆* mRNA
WT (GAL4)	100	100	1.7	2.51 ± 0.12
*gal4-147∆768*	51.7	118	2.5	2.96 ± 0.08
*gal4-238∆768*	75.6	63.8	1.8	1.60 ± 0.06
*gal4-763∆851*	19.1	47	11.5	1.79 ± 0.21
*gal4-238∆851*	28.7	8.9	10.9	0.223 ± 0.01
*gal4-848∆*	85.1	5	22	0.125 ± 0.2
*gal4-844∆*	46.6	4.8	22.8	0.121 ± 0.21
*gal4∆*	0	0.4	ND	0 ± 0.01

*a1* transcript levels from *GAL1pro*::*a1* as determined by qRT-PCR in *SIR4* and *sir4*∆ strains upon GAL induction and media switch from noninducing (raffinose) to inducing (galactose) carbon sources. All *a1* expression values were normalized to *ACT1* mRNA and further to the *a1/ACT1* mRNA ratio in *HMRa1 sir4*∆ strains. Each mRNA expression number represents the average and SEM for three biological replicates.

a DNA binding affinity of *gal4* mutants calculated based on filter binding assay described in Ma and Ptashne (1987).

## Discussion

### Steric occlusion was a major contributor to the mechanism of gene silencing

To date, the T7 RNA polymerase assay represents the most stringent test of the steric occlusion model for several reasons. First, the assay used a transcription protein rather than a simple DNA binding protein as a quantitative test of the Sir protein transcriptional repression mechanism. Second, the assay provided a strong challenge to Sir protein repression because within 2 hr of induction, the level of T7 RNA polymerase was comparable to the level of RNA polymerase II. Yet these cells had only a single T7 promoter, in contrast to thousands of targets for RNA Pol II. Therefore, the T7 polymerase transcription assay stringently measured how effective steric occlusion was at blocking transcription by an RNA polymerase present at a concentration more than 1000-times that of its target sequence. Third, because of its prokaryotic origin, T7 polymerase offered immunity from potential complications resulting from interactions among eukaryotic chromatin proteins.

Sir-based silencing was a significant impediment to T7 polymerase transcription (Figure 2 and Figure 3). The difference between the T7-mediated transcription of *HMR T7pro*::*a1* in *SIR4* *vs.* *sir4*∆ cells was 200-fold at 2 hr after induction, although somewhat lower at longer time points. A 200-fold repression of T7 RNA polymerase due to Sir proteins was in reasonably close quantitative agreement with the 1000-fold magnitude of repression of RNA Pol II by Sir proteins at *HMRa1* and might be accountable by differences in mRNA half-lives.

By 8 hr of induction, a gradual increase in the low-level expression of *a1* transcripts from the *T7pro*::*a1* in *SIR4* cells was detected (Figure 3B). The cause of this low-level escape from silencing was unclear, but the vast excess of T7 RNA polymerase offered a simple possibility. During each cell cycle, silenced chromatin has to be replicated at *HMR*. At least one and probably both chromatids have to assemble new Sir protein complexes. Therefore, replication could be a target of opportunity when the chromatin at the T7 promoter would not yet be completely decorated by Sir proteins and, hence, available to T7 RNA polymerase. The higher level of expression from the T7 promoter in *SIR4* cells at steady state might reflect the additive effect of many successful competitions between T7 RNA polymerase for its promoter over the binding of Sir protein complexes to the chromatin on that promoter.

The issue of what happens to Sir proteins on *HMR* at the later time points after induction of T7 RNA polymerase, when *a1* RNA transcripts were detected, is not fully resolved. ChIP data were consistent with Sir proteins remaining associated with *HMR T7pro*::*a1* in *SIR4* strains even when low-level transcription occurred (Figure 4). However, we could not exclude the simpler possibility that the cells that produced the *a1* transcripts were a different subset of the culture from the cells that contributed this ChIP signal of Sir proteins at *HMR*. It is possible that in batch culture most cells had fully silenced *HMR T7pro*::*a1* and those cells drove the ChIP signal, whereas a small population of cells in the culture, if derepressed, could account for the small *a1* transcript signal.

The mechanism of Sir-based silencing was largely independent of any special feature of the native transcription machinery. Occlusion of a specific eukaryotic transcription factor or coactivator would have no effect on blocking transcription by T7 RNA polymerase. It remains unclear whether T7 RNA polymerase was physically prevented from binding its promoter in silenced chromatin, or whether the 200-fold difference in repression between *SIR4* and *sir4∆* strains reflects a mixture of reduced polymerase binding and reduced postbinding events. We have not been able to produce an epitope-tagged T7 RNA polymerase that still functions as a polymerase. Existing antibodies, although serviceable for immunoblots, have not provided sufficient sensitivity in ChIP assays to test whether Sir-based inhibition of promoter binding could account for the full magnitude of the effect.

Histone deactylation as a mechanism of gene repression is shared among many taxa. However, the use of Sir proteins as structural components of heterochromatin is specific to yeast. Surprisingly, in analogous experiments using Gal4 and T7 polymerase to investigate repression of transcription by *Drosophila* polycomb complex, Polycomb blocks Gal4 activation but not T7 polymerase from productive transcription in flies (McCall and Bender 1996; Fitzgerald and Bender 2001).

### No evidence of RNA polymerase II in silenced chromatin

Our results were incompatible with the downstream-inhibition model for silencing in yeast and call into question the data supporting that model. Specifically, the critical prediction of this model is that there would be a position near the promoter of α1, where RNA Pol II is found within Sir-silenced heterochromatin bound on the template and with the two strands of the template held apart in what is equivalent to a paused transcription bubble. The nearly identical cleavage pattern of the permanganate-labeled DNA from this region in *SIR4* and *sir4* cells indicated a lack of any position at *HML****α****1* in silenced cells at which RNA polymerase II was stalled (Figure 1). Likewise, the pattern of permanganate reactivity was indistinguishable between *HML****α****1* silenced by Sir proteins and *HML****α****1* repressed by Tup1/Ssn6 corepressor acting through the a1/***α****2* repressor (Strathern *et al.* 1981; Komachi *et al.* 1994) . The mechanism of repression by a1/**α***2* through Ssn6/Tup1 does not involve a stalled polymerase, but rather acts at the level of transcription initiation (Parnell and Stillman 2011). It should be noted that, in contrast to other organisms, there has not been a paused polymerase in yeast detected by the permanganate assay. Hence, formally there was no direct positive control for this result. Nevertheless, together with the other results presented here, the most parsimonious interpretation is that the mechanism of silencing is one that operates prior to any engagement by RNA polymerase.

ChIP-exo-seq (Rhee and Pugh 2013) and nascent transcript-seq datasets (Churchman and Weissman 2012) also contained no evidence of RNA PolI II at silenced *HML*. The lack of a stalled RNA Pol II in silenced chromatin led us to conclude that the primary repressive mechanism of Sir protein–mediated silencing acts before DNA melting in the transcription cascade. Previous reports of ChIP (Gao and Gross 2008) and ChIP genome-wide array data (Steinmetz *et al.* 2006) that inspired the downstream-inhibition model were likely misled by the relative shearing resistance of silenced chromatin (Teytelman *et al.* 2009) resulting in artifactual precipitation of *HML* and *HMR* with RNA polymerase engaged on flanking genes in inadequately sheared chromatin.

In silenced chromatin, there remained a five-fold difference between the 200-fold repression of T7 RNA polymerase and the 1000-fold repression of transcription of the native *HMRa1* that has not been accounted for. We cannot exclude the possibility that in a small subset of cells RNA polymerase II occasionally does engage the *a1* promoter at *HMR*, perhaps when the chromatin is replicated, yet fails to elongate all the way through silenced chromatin. There is a precedent for a different mechanism of epigenetic silencing that blocks transcriptional elongation as has been shown for methylated genes in *Neurospora* (Rountree and Selker 1997). If so, then our data indicated that there would be no unique arrest point of such polymerase molecules within the detection sensitivity of the assay. A simpler model to explain the discrepancy between the 200-fold repression of T7 RNA polymerase and 1000-fold repression of transcription of the native promoters at *HML* and *HMR* might be differences in the half-lives of the different RNAs, which to date have not been evaluated.

### Implications of the Gal4 activator escape from silencing at HMR

The results of the experiments with the *HMR GAL1pro*::*a1* allele highlight that a simple steric occlusion model alone cannot explain all aspects of the mechanism of transcriptional silencing. Gal4-activated transcription was repressed, at most, two-fold due to Sir proteins (Figure 6).

The affinities of Abf1, Gal4, and T7 polymerase for their binding sites appear to be similar (in the low nM range) (Taylor *et al.* 1991; Ujvári and Martin 1997; Beinoravičiūtė-Kellner *et al.* 2005). Yet, T7 RNA polymerase and Abf1-dependent activation are dramatically repressed, but Gal4 can both access its binding site and promote transcription in the presence of Sir proteins.

In addition, Gal4 was, by inference, able to recruit coactivators to silenced regions *in vivo*, in contrast to *in vitro* data claiming activator-interference is a primary silencing mechanism (Johnson *et al.* 2013). In the first hour of galactose induction, there was no difference in transcription at *HMR GAL1pro*::*a1* between *SIR4* and *sir4*∆ strains (Figure 7). The identical transcript induction kinetics in *SIR4* and *sir4*∆ strains over the first hour suggests that Gal4 had already occupied its binding site in cells of either genotype before induction began. Moreover, the rate of mRNA production in the two strains was similar for that first hour. Hence, by these criteria, Gal4 was largely immune from Sir-based silencing. At later time points there was a consistent approximately two-fold higher level of transcripts in *sir4∆* than in *SIR4*. The reason for this difference remains unknown. It is striking to us that Gal4 appeared to be largely immune to silencing given that the expression level from *HMR Gal1pro*::*a1* was only 2-fold to 2.5-fold greater than achieved by the native *a1* promoter at wild-type *HMR* in *sir4* cells. A recent article concludes that transcription factors that are weak activators are sensitive to Sir-based silencing, whereas those that are stronger activators are not (Wang *et al.* 2015). It would be striking if such a small difference in activator strength could explain the difference in silencing sensitivity of Abf1 relative to Gal4.

Conceptually, being able to determine whether Rap1 occupies its binding site at *HML* and whether Abf1 occupies its binding site at *HMR* (McBroom and Sadowski 1995) would provide useful information regarding the mechanism of silencing. Unfortunately, the quality of the reagents needed for this assessment, combined with the relative shearing resistance of *HML* and *HMR* (Teytelman *et al.* 2009), and the inaccessibility of key positions within *HML* and *HMR* to antibodies (Thurtle and Rine 2013) have, to date, prevented a clear resolution.
